# Next-Generation Biomaterials: Design Strategies, Clinical Translation, and the Rise of Intelligent Therapeutic Platforms

**DOI:** 10.1021/acsomega.5c06034

**Published:** 2025-09-29

**Authors:** Gayathri Chakrapani, Vishnu Vijay Kumar, Mina Zare

**Affiliations:** † Academy of Scientific and Innovative Research (AcSIR), Ghaziabad 201002, India; ‡ Department of Biochemistry and Biotechnology Laboratory, 29823 CSIR - Central Leather Research Institute, Chennai 600020, India; § Structural Engineering, Division of Engineering, 167632 New York University Abu Dhabi (NYUAD), PO Box 129188, Abu Dhabi 129188, United Arab Emirates; ∥ Department of Food and Nutrition, Faculty of Agriculture and Forestry, 3835 University of Helsinki, 00014 Helsinki, Finland; ⊥ Helsinki Institute of Sustainability Science (HELSUS), 3835 University of Helsinki, 00014 Helsinki, Finland

## Abstract

Biomaterials are emerging as dynamic, programmable systems designed to interact with biological environments precisely and purposefully. This review highlights cutting-edge biofabrication strategies for nanostructured scaffolds, stimuli-responsive polymers, bioresorbable metallic and polymeric implants, and smart drug-delivery platforms, linking design principles to functional performance and clinical translation. We discuss design strategies that are converging with artificial intelligence and machine learning to accelerate material discovery, enable property optimization, and advance innovations from laboratory research to clinical use. Persistent scalability, reproducibility, and regulatory approval challenges are assessed alongside emerging, sustainable solutions that prioritize clinical viability. By integrating insights from materials science, bioengineering, and translational medicine, this work envisions intelligent therapeutic platforms capable of delivering safer, more personalized, and future-ready biomedical interventions.

## Graphical abstract

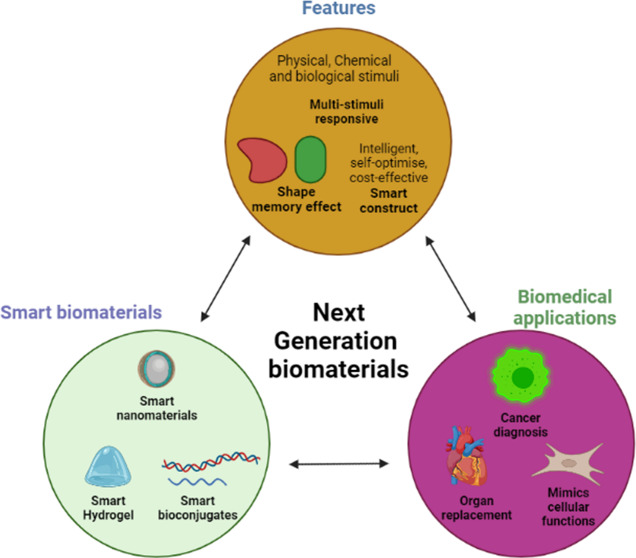

## 1 Introduction

Recent developments in biomaterials have nurtured the clinical strategy of restoring or replacing the normal function of diseased or deformed tissues/organs.^1^ The selection of biomaterials considers several aspects, such as physical strength, mechanical strength, and physical qualities. Although the use of various materials to restore function, improve functionality, and increase the reproducibility of biomaterials, stability and biodegradability are essential for long-term use.^2^ Scientists are engineering “smart biomaterials” with enhanced site-specific functionality, optimized biodegradability, improved biocompatibility, and greater strength. These materials aim to reduce cytotoxicity and accelerate functional recovery in medical applications.^3^ The design includes the clinical condition fixed by understanding the mechanism of the disease to engineer the cell-material contact, signaling progress, and response behavior, which integrates the understanding of the cellular mechanism and materials science.^4^ The design of biomaterials to improve compatibility and reduce toxicity while increasing biodegradability is essential. Hence, a nature-inspired design is preferred for the delivery of therapeutics. The biomimicking, bioinspired, and bioactive architecture is similar to that of nature-associated systems in the biomedicine field. The design has better self-healing capabilities, mechanical strength, and adaptability to the host system.^5^ Biomimetic material synthesis refers to procedures similar to those found in nature, such as employing live organisms or extracting components from organisms to make inorganic systems biochemically and structurally identical with in vivo systems. Structural features play a significant role in the mechanical strengthening of biomaterials.^6^ The dimensional feature involves adaptation of the biomaterial in the host system. The design of biomaterials in the 10–9th percentile of a meter, which are more specific to disease locations, is nanoscale. The scaling range varied from 0.1 to 100 μm for microscale biomaterials. Furthermore, biomaterials with dimensions above 100 μm are macroscale biomaterials.^7,8^ It is imperative to analyze the interfacial interactions at some dimensional scales to learn more about how a material behaves in both single- and multiasperity interactions and to increase the dependability of biomaterials. The variation at the dimensional scale and their interactions with varying cellular behaviors are coordinated by molecular gradients, localized substrate rigidity, nanoscale network structure, microscale spatial configuration of cells in relation to one another, and functional interactions across tissues.^9^

Biomaterial design plays a significant role in treatment strategies and has enormous effects on the rapid healing and fixation of deformations.^10^ The era of bioprinting is constantly increasing from 2-dimensional (2D) to 4-dimensional (4D) methods, beyond which functional organ printing, microfluidic chips, artificial intelligence (AI)-based sensors, imaging techniques, nanocoated stents, and valves are emerging to improve treatment strategies.^11^ Recent interest in the fabrication of biomaterials via three-dimensional (3D) bioprinting has given rise to flexibility in the bioprinting of the tissues of interest through the infiltration of living cells, aggregates, bioactive molecules, immune cells, and growth factors for the assembly of bioprinted tissues. This improves the reproducibility and mimicking of the tissues. Natural, semisynthetic, and synthetic polymeric substances are used for the bioprinting support. Cellular infiltration is improved and printed using collagen-I solutions that can infiltrate.^12^ Zhang et al. developed a gut tube that can enter cells via 3D bioprinting. This approach creates a matrix that can adapt to a heterogeneous cell population.^13,14^ The role of biomaterials in cell regeneration is to construct artificial skin to replace the deformed skin region due to ulcers or burns.^10^ Biomaterials also bring technological improvements in the design and development of new devices to detect and analyze samples. Innovative biomaterials are also being designed, specifically those triggered based on certain activation conditions, such as pH, redox, and self-activation.^15^

A new class of biomaterials has evolved from wet laboratories, showing promising applications in cancer treatment, tissue regeneration, and other therapeutic areas. However, the traditional “trial-and-error” approach to biomaterial fabrication limits the speed and precision needed for clinical translation.^16^ To overcome these challenges, informatics and computational modeling have emerged as critical tools for understanding biomaterial function and structure interactions, enabling more precise formulation and synthesis. AI, with its capacity to mimic human cognition through complex problem-solving and decision-making, offers a transformative approach to accelerate biomaterial discovery and optimize their properties. Focused on AI, machine learning (ML) algorithms can automatically recognize patterns from large data sets, predicting biomaterial characteristics and performance, thereby shifting biomaterial research from empirical methods to data-driven design.^17^ This review focuses on the integration of AI-driven strategies within biomaterial development, aiming to bridge the gap between laboratory innovation and clinical application. We discuss (Section 2) different biomaterial fabrication methods and their biomedical applications; (Section 3) clinical applications of biomaterials; and (Section 4) next-generation biomaterials shaped by AI and computational tools. By explicitly addressing the need for AI-enhanced biomaterial design, this work highlights a pathway toward more efficient, reproducible, and clinically viable therapeutic platforms.

## 2 Different Biomaterial Fabrication Methods and Their Biomedical Applications

The design of biomaterials requires the integration of physicochemical aspects that match the in vitro conditions, facilitating low cytotoxicity.^18^ Biomaterials have been a needful field of research for dental repair, stents or valves, wound healing scaffolds, orthopedic implants, cranioplasty implants, and other tissue engineering implants; the need to fix the clinical conditions led to several biomaterials. The unique features of biomaterials include bioactivity, tissue-like mechanical properties, osteoinduction/osteoconduction/osteogenesis properties, cytocompatibility, and biodegradability for medical applications.^19^ Figure 1 depicts the different types of evolved nanomaterials, ranging from extra dimensions for diverse applications.

**Figure 1:**
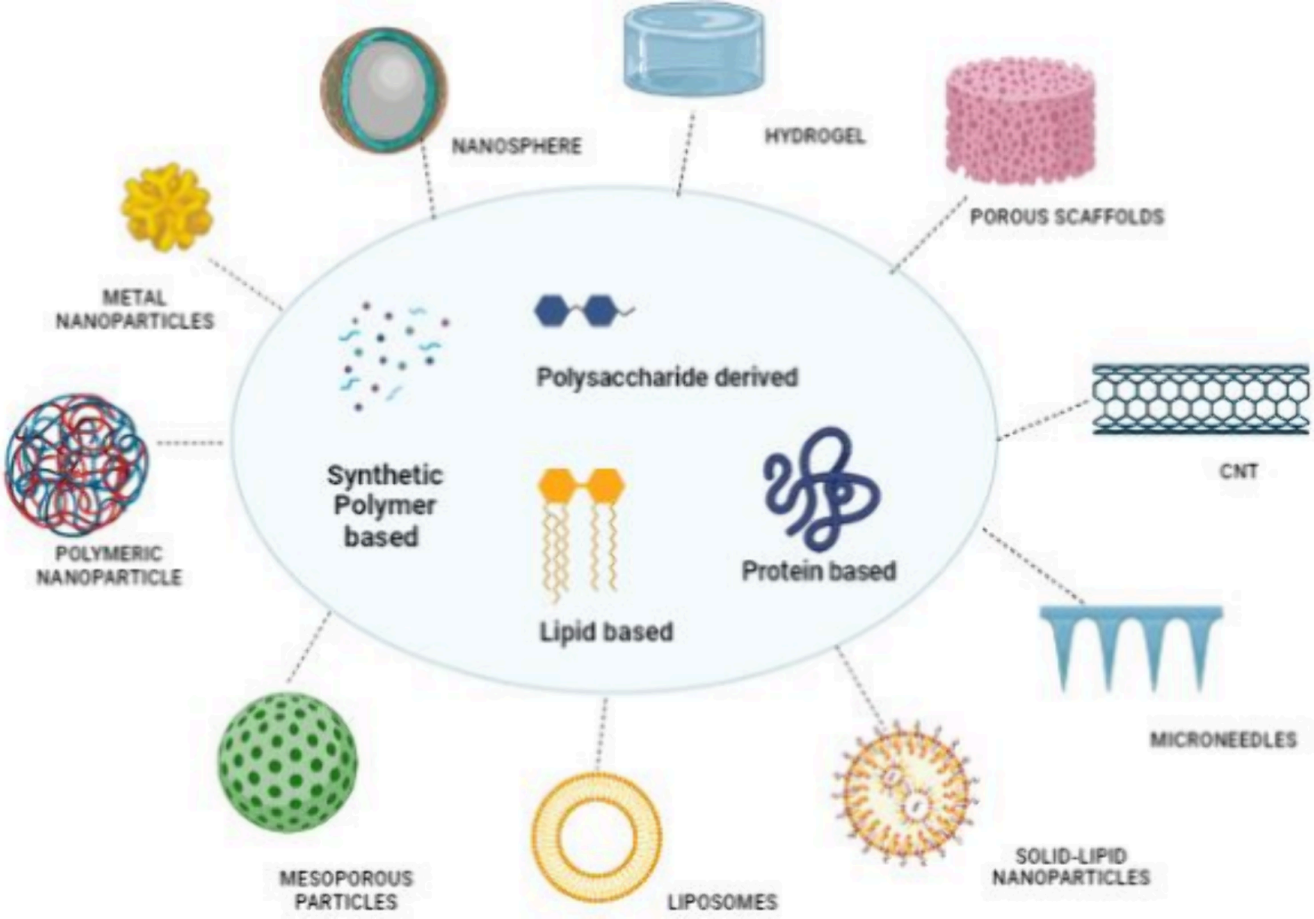
Diverse biomaterial-based delivery platforms are categorized by origin and composition. The central classification includes polysaccharide-derived, protein-based, lipid-based, and synthetic polymer-based systems. And various architects, nanospheres, hydrogels, porous scaffolds, carbon nanotubes (CNTs), microneedles, solid lipid nanoparticles, liposomes, mesoporous particles, polymeric nanoparticles, and metal nanoparticles. These platforms are widely utilized in drug delivery, tissue engineering, diagnostics, and regenerative medicine applications.

### 2.1 Nanoparticles: Convenient and Diverse Applications

Nanoparticles (NPs) and their diverse applications have attracted the interest of many researchers in drug delivery in recent years. Several nanopreparations have been disseminated in drug development and design to improve the efficacy, safety, and tolerance of integrated pharmaceuticals.^11^ Owing to their size, the surface-to-volume ratio and charged NPs initiate cell adhesion and interaction. One of the familiar forms of NPs is spherical; however, various shapes, such as rods, cubes, polygons, sheets, and plates, have been synthesized for various applications.^20–22^ The properties of the nanocubes and rods are better than those of the other shapes, probably because of the exposed planes and oxidation levels. A study of surface crystalline faces revealed that unstable surfaces consume less energy to produce oxygenated pores, which is related to the bactericidal activity of the NPs.^23,24^ The form is crucial even among NPs with comparable surface areas because characteristics with a high atom packing density increase responsiveness. They alter biological applications via different mechanisms.

NPs differ from other materials in their ability to inhibit microbial development, and as a result, they have been used to combat infectious diseases.^25^ NPs inside cells interact with the microbial cellular machinery from enzyme inactivation to protein deactivation and induce oxidative stress and electrolyte imbalance, which modify gene expression levels.^26^ One primary inhibition mechanism involves the generation of reactive oxygen species, which cause oxidative stress either through interaction with Coenzyme (Q10) or through its function in the electron transport chain (ETC) to act against bacteria to damage lipids, proteins, and DNA.^27^ The surface charge influences the antimicrobial activity by bridging the interactions of the NPs with the microorganisms. The microorganism’s cell wall is negatively charged, and the positively charged NPs are electrostatically attracted to the bacterial layer. This infuses NPS into the structure, disrupting the cell wall and contributing to its bactericidal activity. The concentration of ions is responsible for toxicity in cells.^28^ NPS that engage with the bacterial cell wall form a focused source of constantly released ions, increasing cell toxicity.^29,30^ The high ion concentration also aids penetration within the cell membrane; dissolution depends on the size and shape of the NP. As a result, NPS dissolution is limited around the bacterial cell membrane, with the kinetics of dissolution depending on the size and shape of the NPs.^31,32^ The interaction and mechanism of action of the nanomaterial with bacterial cells and its strategies used to target bacteria are shown in Figure 2.

**Figure 2:**
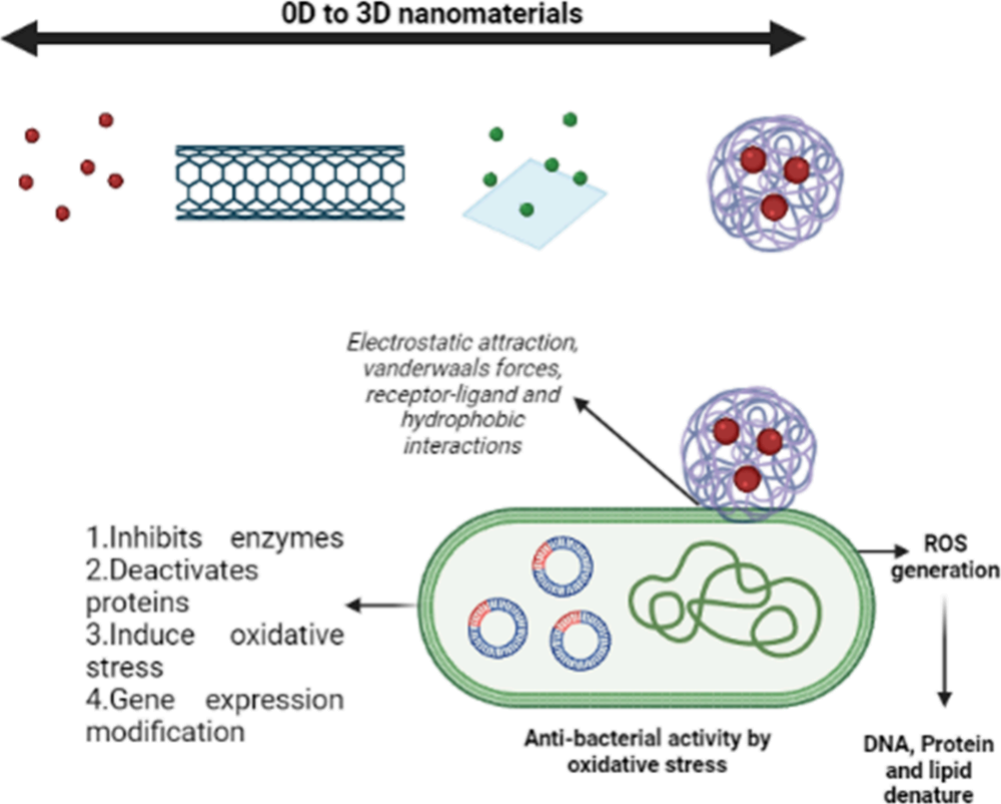
Varying scales of nanomaterials from zero dimensions to three dimensions. The interaction of a nanomaterial with a bacterial cell and its mechanism of action to inhibit bacterial growth are demonstrated above.

In drug delivery, NPs can be administered and targeted to specific tissues or organs and then employed in noninvasive real-time cell observation and tracking via various imaging techniques, therapeutic drug nanocarriers, or scaffolds to guide the creation of new tissue.^33^ Gold, iron, titanium, zinc oxide, cadmium, selenide, and carbon NPs are classic chemical NPSs exploited in regenerative medicine.^34^ Nanomaterials are versatile, enabling them to integrate functionally related components into a single unit and allowing tremendous advancements in imaging, sensing, and structural technologies. They are classified as carbon-based NPs, metal NPs, ceramic NPs, lipid NPs, polymeric NPs, or semiconductor NPs, and are synthesized via bottom-up and top-down synthesis approaches.^35^

The synergistic assembly of NPs in the lipid bilayer system is facilitated by the synthesis of metal oxide NPs with lipid particles. The self-assembly of NPs in the interior of lipid bilayers is understood via coarse-grained molecular dynamics (CGMD) simulations.^21^ PEGylated liposomal doxorubicin (PLD) has diverse drug delivery applications. The vesicle diameter of PLD is 105 nm with a PEG surface coating. PEG lowers muco polysaccharide (MPS) uptake by preventing opsonization by plasma proteins.^36^ The treatment of AIDS-related Kaposi’s sarcoma with liposomal daunorubicin cysts, 45 nm in size, has been approved. Abraxane (Nab-paclitaxel) is a 130 nm NP version of albumin-bound paclitaxel.^37^ It has been approved for the treatment of breast cancer and is currently being studied in clinical trials for other indications. The pharmacokinetics of nab-paclitaxel and cremophor-paclitaxel have been compared.^37,38^ The plasma clearance and distribution volume of nab-paclitaxel were significant. Abraxane (Nab-paclitaxel) is a 130 nm NP formulation of paclitaxel that is albumin-bound. It has been approved for breast cancer treatment and is currently being examined in clinical trials for other indications. The pharmacokinetics of nab-paclitaxel and cremophor (Taxol)-paclitaxel were compared. The plasma clearance and distribution of nab-paclitaxel are more significant.^39,40^

The advancements in nanotechnology in medicine, drug delivery, and sensing have leveled up to another extent in vivo through nanobots.^41^ Nanorobots are devices composed of units with nanometer-scale dimensions. Nanorobotic applications have been extensively used in various medical applications, including microbiology, hematology, surgery, and dentistry. Drug delivery systems have gained interest because of their ability to deliver drugs precisely to the target site, avoiding the systemic effects of drugs, helping them play a vital role in transporting and delivering cells, wound healing treatment, cancer detection and biopsy, real-time imaging, biosensing, and retention of payloads in the gastrointestinal tract.^42^ In addition, they can deliver drugs at a higher speed and precision to the targeted region. Adding the motile microgrippers in the nanorobots helps reach the out-of-reach areas of human systems, aiding noninvasive surgery.^43^ Furthermore, the nanorobot travels to sites, collects tissues, captures the disease condition, enters the bloodstream, and reports the blood contents to health care professionals as a biosensing tool.

### 2.2 Biomedical Applications of 2D Materials and Quantum Particles

Nanotechnology has been widely accepted as a potential technique for addressing several healthcare conditions, drug delivery, and disease diagnosis.^44^ The structural features, distinctive architecture, physical and chemical properties, and pharmacological impact of 2D biomaterials make them diverse. It possesses novel dimensional benefits with planar topology, which has led to exponential research in many scientific approaches.^45^ The field originated with the advancement of a single layer of graphene from graphite in biomedical applications; it was successively followed by hundreds of varieties of thin 2D nanomaterials possessing bioapplications.^46^ Two-dimensional biomaterials are fabricated by size reduction to a few layers or a single layer. The synthesis strategy focuses mainly on the composition modification of the nanomaterial surface through (i) top-down synthesis, (ii) bottom-up synthesis, and (iii) wet-chemical solvothermal reaction methods.^18^

The general structural architecture has an ultrathin thickness and a lattice with distinct planes. Fabrication focuses on the crucial aspect of stability during the physiological explosion. They tend to aggregate and lead to a poor strength. Therefore, proper surface functionalization is required for stable strength.^21^ Interactions through van der Waals forces acquire the π–π interactions, hydrophobic/hydrophilic interactions, electrostatic interactions, and receptor–ligand binding with biomolecules.^29,47^ The self-assembly process of biomolecules occurs through various interactions between the biomolecules. They self-assembled on different templates and substrate media. Figure 3 illustrates the biomedical and technological applications of quantum dots.

**Figure 3:**
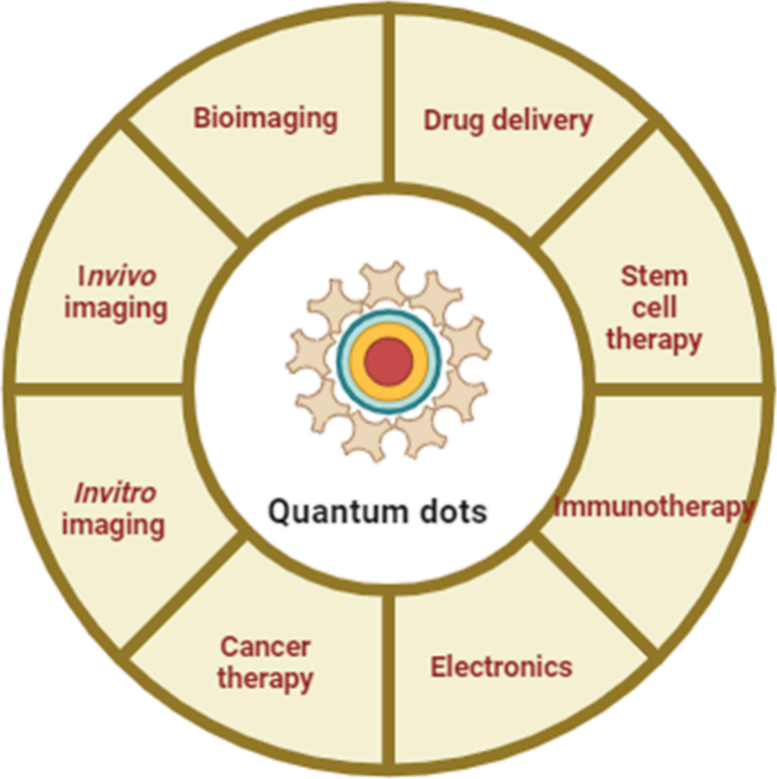
An overview of the diverse biomedical and technological applications of quantum dots, including bioimaging *(in vivo and in vitro imaging),* drug delivery, stem cell therapy, immunotherapy, cancer therapy, and electronics.

Moreover, 2D nanomaterial applications include biosensors, bioimaging, therapeutic drug delivery, anticancer theranostics, and regenerative medicine.^48^ 2D biomolecules based on nucleic acids, proteins, and peptides follow a definite architecture.^49^ Another essential tool of 2D materials that has gained attention is semiconducting NPs [quantum dots (QDDs)]. They have a dimensional range of 1–10 nm, with simultaneous targeting and imaging potential in drug delivery and pharmacological and biological applications.^50^ Quantum dots have low photobleachability and are potential fluorescent probes for various tagging studies. The size influences the fluorescence wavelength and optical properties of the quantum dots. In other words, a spectrum of colors can be radiated from a single component by varying the dot size. QDs attached to biomolecules exhibit binding selectivity without compromising the ability of the compounds to mark specific biological proteins.^51,52^ Figure 2a illustrates the quantum dots’ biomedical applications.

### 2.3 Extracellular Vesicles: Potential Drug Delivery

The more intricate type of liposome with a biological origin, known as extracellular vesicles (EVs), has emerged. EVs are nanosized particles covered in a sophisticated lipid bilayer. Exosomes and microvesicles are released from living cells independently or in response to external stimuli. The multivesicular bodies of the endosomal system are where exosomes are produced.^53^ Furthermore, polypeptide-based targeted medicine can be a promising tool for cancer therapy. Polypeptide-based medicines are dynamically stabilized with various acids, ROS, and other substances through conjugation, lipid–liquid interactions, π–π stacking, and the formation of micelles.^54^

Exosomes are released when the multivesicular body and plasma membrane fuse. Compared with liposomes, which have phosphatidyl inositol, phosphoglycerol, phosphatidyl ethanolamine, and phosphatidylcholine, along with their functionalized lipids under anionic, cationic, and Zwitterionic classifications, EVs predominantly contain glycosphingolipids, sphingomyelins, phosphatidylethanolamines, phosphatidylserines, phosphatidylcholines, and phosphatidyl lipids. EVs have diverse applications in site-specific drug delivery and are extensively utilized in cancer treatments.^55^ A thorough examination of EVs as drug delivery vehicles may provide exciting insights that can be used to synthesize particles because of their biological sources and biocompatibility. Examples include tailoring the lipid composition or functionalizing it with different bioinspired moieties to create next-generation liposomal drug delivery methods with improved efficacy and cytocompatibility. The homing of vesicles to the target tissue, appropriate absorption in target cells, and effective therapeutic payload delivery could all benefit from understanding the EV domain.^56^

### 2.4 Lignin Nanocarriers

Machago et al. reported that biodegradable delivery vehicles made of lignin nanocarriers are desirable for stem injections against the deadly fungus Esca. However, they are promising candidates for treating other antifungal plant diseases.^57,58^ The development of techniques for drug delivery to ovarian cancer (OC) was discussed by Wang et al. They highlighted that clinical trials using folate-drug conjugates and antibody-drug conjugates (ADCs) in patients with advanced-stage and chemoresistant OC have demonstrated that localized administration of toxic drugs can significantly increase their tumor cell selectivity, tumor absorption, and antitumor efficacy. The dose-limiting toxicity of targeted drug complexes, partly caused by their poor tumor selectivity and poor drug release control, still frequently restricts their clinical usefulness. The dual-targeting approach can improve tumor penetration, resolve concerns about tumor heterogeneity, and increase OC cell selectivity and absorption, each of which has decreased off-target damage.^59^

### 2.5 Recent and Novel Application of Hydrogels

Hydrogels are hydrophilic polymeric networks that are more attracted to water as a dissolution medium with interconnected cross-links.^60^ They are naturally soft and nontoxic, and their high water content makes them feasible to handle. This makes them injectable, biocompatible, biodegradable, mucoadhesive, and programmable bioadhesive characteristics that offer potential in various applications.^61,62^ These characteristics make hydrogels appealing materials for tissue regeneration and drug delivery to specific body locations. The polymeric substances swell between the hydrogels’ polymeric network. This higher ‘hydro’ content in the ‘hydrogels’ makes them more desirable for biological applications.^63^ The hydrogel architecture varies at the scale level: at the macroscopic scale, the hydrogel architecture involves the size and nature of the gel, as well as the interconnected networks and pores present within it.^60^ The visual design is furthered by the mesh size of the gel and the binding of small molecules in the polymeric network. Hydrogels have been used in clinical trials to modulate the release of therapeutic substances over space and time. Size and binding are related to the release of drugs in the human body.^64^ The hydrogel dimensions and pore structure are part of the macroscopic design. Hydrogels can be either nonporous or have macropores ranging from 10 to 500 μm in size. The mesh size (the distance between polymer molecules in the network) can be adjusted from 5 to 100 nm.^65^ Therapeutics can interact with polymer chains on a molecular scale through various methods; a covalent bond is depicted in Figure 4.

**Figure 4:**
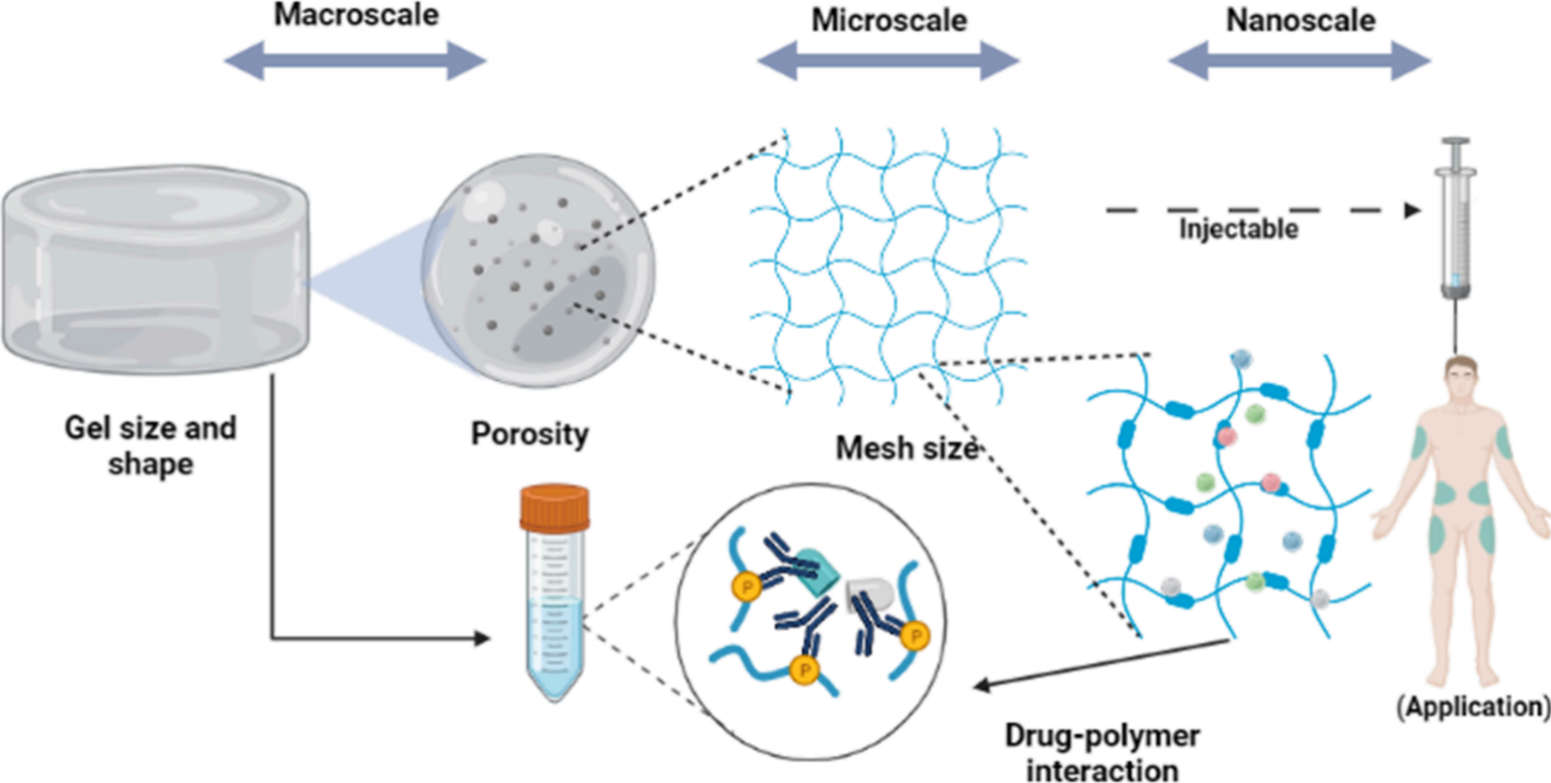
Varying dimensions of the hydrogels in different applications. The porosity, gel shape, and size are evaluated on the macroscale. In the microscale dimension range, the mesh size range is evaluated. In the nanoscale range, the polymeric interactions with the drug are evaluated.

The biocompatibility and adaptability of these materials have attracted increased interest in tissue engineering and drug distribution applications.^66^ Hydrophilic polymers are chosen on the basis of their ability to interfere with the host microenvironment. The design of the formulation is based on the ability to polymerize and homogenize in the dissolution medium, with the fluid amount embedded and retained in the polymeric chains.^67^ The polymeric network immersed in water attains its equilibrium state with two reactive or opposing forces: a thermodynamic force to mix and a retractive force to oppose. The hydrogel structure can be elucidated via two major theories: (1) equilibrium-swelling theory and (2) rubber elasticity theory.^68,69^ The hydrogel can recover to a normal state from the applied stress up to one-fifth of the deformation range. The hydrogel moieties do not possess ionic bonds between the polymeric chains. They have varying sizes of the porous structural network in the hydrogel, classifying them as microporous, nanoporous, or macroporous.^70–72^ The networking between the polymers is initiated by thermal, chemical, or photobased processes. Stimulus-sensitive hydrogels specifically interact with specific biomolecules, such as proteins, glucose, enzymes, genes, or environmental conditions^73^ like the swelling matrix of a hydrogel, which is based on its flexible or elastic outer layer properties. In addition, pore-forming ability is a determining factor for cell adhesion, which is a primary concern in the design of biomaterials.^64^ Injectable hydrogels are promising alternatives for noninvasive surgical conditions. Hydrogels are effective biosensors that can be used for surface modification.^74^

Synthetic hydrogels are prepared from polymers such as poly­(hydroxyethyl methacrylate) (PHEMA), poly­(vinyl alcohol) (PVA), polyethylene glycol (PEG), polylactic acid (PLA), and poly­(ethylene oxide) (PEO), which are among the few neutral synthetic biomaterials used based on molecular weight for various applications. PEG is the only widely utilized polymer, as the immune system subtly recognizes it and does not accumulate proteins on the surface. Natural polymers are further classified into protein-based, polysaccharide-based, and decellularized polymers. Nanocomposite-based hydrogels are synthesized from carbon-based materials, metal oxides, and inorganic material-based polymers.^14^ The clinical translation of hydrogels is critical because of their high water content; biomaterials may undergo stability issues and require specific storage conditions with constant monitoring of time and temperature. Regulatory concerns frequently hamper the development of hydrogel delivery systems and the high cost of commercialization.^75–77^ Because a hydrogel that releases a medication or encapsulates stimulant cells is controlled as a drug combination, its regulatory approval process is typically longer than that of a scaffold with no payloads. Because patent rights are limited in duration, a longer approval process can impede the commercial viability.

Hydrogel fabrication technique largely depends on their behavior during and after the bioprinting. Their network structure, cross-linking density, degradation rate, and overall strength depend on the fabrication method, and these factors decide whether a hydrogel can print smoothly, hold its shape, and still support living cells. The stable network is usually based on the cross-linking mechanism: either physical or chemical cross-linking. Wherein, the polymeric network is bound and allows the structure to be stable. Hybrid strategies that mix the two in order to get enhanced structural stability.^78,79^ In the aspects of printability, three properties are especially important: gelation speed, shear-thinning behavior, and yield stress. If a gel solidifies too quickly, it clogs the nozzle before the printing is complete; if it gels too slowly, the printed filament collapses or spreads out. The sweet spot is often achieved by combining fast but reversible ionic bonds with slower, permanent covalent bonds. Likewise, shear-thinning behavior allows the material to flow easily through the nozzle under pressure and then recover once deposited. Further, if the yield stress is too low, the printed strands will merge and lose definition; if it is too high, extrusion becomes difficult and can damage cells due to excess pressure.^80^ Physical approaches, like freeze-thaw cycling of PVA or calcium ion cross-linking of alginate, are simple and cell-friendly but are subject to weak mechanical strength. Chemical methods, such as UV light-triggered GelMA or enzyme-mediated silk fibroin–gelatin gels, result in stronger and longer-lasting scaffolds but require more careful control to avoid harming cells.^81^ Irrespective of the fabrication method, the stiffer gels provide better structural fidelity but can restrict nutrient transport and reduce cell viability, while softer gels facilitate cell growth but often collapse after printing.^82^ Several promising solutions are emerging. Hybrid systems that combine ionic and covalent bonds give immediate print fidelity, along with long-term strength. Dynamic hydrogels with reversible covalent bonds can “heal” after deformation, offering both robustness and cell-friendly softness. Stiffness-gradient scaffolds allow strong support in load-bearing areas while keeping softer niches for embedded cells.^83^ Above all, advanced fabrication technologies such as extrusion bioprinting, stereolithography, and microfluidic gelation, when paired with computational modeling and AI optimization, now allow researchers to predict and tune properties like gelation speed, yield stress, and degradation rate. This integration is helping to finally solve the classic challenge of balancing structural stability with biological compatibility, making it possible to design hydrogels that print well, hold their shape, and still keep cells alive and functional.^84^ They contain reversible covalent bonds, and they are Increasingly, these fabrication workflows are integrated with computational modeling, AI, and ML to predict and optimize gelation kinetics, shear-thinning behavior, yield stress, and degradation rates, enabling the rational design of hydrogels that reconcile the inherent trade-off between structural stability and the softness, porosity, and bioactivity required for high cell viability in translational biomedical applications. Table 2 lists the methods of hydrogel preparation in recent times.

**Table 1:** Biomaterials with Their Preparatory Methods and Clinical Applications, Along with the Characteristics of the Biomaterial

biomaterials and their types	method of preparation	in vivo applications	characteristics/remarks	ref
fluorescent dots	chemical synthesis of hydrophobic amino acids with water-soluble hyaluronic acid	bioimaging	greener approach to synthesis	^165^
		drug delivery in tumor cellular systems		
organ model	3D printing of organ models with living cells using the FLOAT method (stereolithography)	replacement of organs	lifelike organ models are created using ultrafast 3D printing	^166^
		fast and rapid printing technology		
“sonothermogentics”	sound, heat, and genetics were combined to develop a noninvasive technology to stimulate certain cell types deep within the brain	to control motor activity in a mouse’s brain	focused ultrasound (FUS) switches on specific mouse neurons by noninvasively activating the TRPV1 receptor. When FUS touches TRPV1 on the surface of mouse brain cells, the receptor opens, allowing calcium ions to enter and activate the neuron	^121^
		noninvasive treatment for neurological disorder patients		
ankle prosthetic	the residual limb is fitted with surface electrodes that monitor electrical impulses in the user’s residual muscles. The electrodes gather and process the related EMG activity when the user contracts their residual muscles, such as flexing their foot. This EMG signal controls the movement of the mechanical limb by pneumatic artificial muscles, which contract and extend using compressed air	prosthetic ankle for intuitive balance correction	a motorized prosthetic ankle’s dEMG control paradigm for postural control	^167^
alginate-based lung sealant	methacrylate alginate and AMA-dialdehyde are used for sealing damaged tissues and regeneration	pleural sealant to treat lung leakage in the treatment of pneumonia and cystic fibrosis	lung leaks caused by surgery, injury, or illnesses like pneumonia and cystic fibrosis can all be treated with a therapeutic patch	^168^
composite hydrogel for bone regeneration	physically blending the components of NBG (amino-modified bioactive glass) and CAG (electrostatically reinforced hydrogel)	bone defect repair and new bone formation	cationic amino-modified bioactive glass (NBG) and anionic gellan gum form an electrostatically reinforced hydrogel (AGG); ionically cross-linked using calcium ions; the nonsfunctionalized bioglass is mixed with gellan gum (AG)	^169^
keratin-fibrinogen (KRT-FIB) hydrogel	human extraction to succinylation to Facile coupling reaction synthesizes KRT-FIB hydrogels	oral tissue engineering	encapsulated HGFs proliferated more effectively when encapsulated in KFH, and HGFs had a diffuse shape when encapsulated in KFH	^64^
lab-grown mammalian tissue	prepared using palnts. Foods are soaked in the nutrients, and cells are grown on the matrix	3D scaffolds for Tissue engineering applications	decellularized plant materials are utilized for tissue engineering applications	^170^
nanodisk	a strong synergy of arrayed radiative coupling and substrate undercut in the visible light spectrum can enable high-performance biosensing	antibodies functionalized nanodisk for Rapid Cancer detection for Point-of-care application	AGNIS (arrayed gold nanodisks on an invisible substrate) as a label-free (no fluorescent labels required), cost-effective, and high-performance platform for molecularly selective exosome biosensing of early stage cancer	^171^
sonobiopsy	standard blood-based liquid biopsy is revolutionized by focused ultrasound-enabled liquid biopsy (sonobiopsy), improving diagnostic sensitivity of glioblastoma-specific genetic alterations.	glioblastoma detection. The characterization is minimally invasive, spatiotemporally controlled, and sensitive.	sonobiopsy breaks down the BBB to a particular site in the brain, allowing tumor-derived DNA to enter the bloodstream and ctDNA to be collected promptly.	^153^
artificial intelligence	a set of processes that allows controlling with computer programming and algorithm to perform tasks	wearable sensor for glucose level prediction	diverse applications in health care applications like imaging, radiology, clinical care and telehealth	^172,173^
		screening colorectal cancer by enhanced image analysis		
		design of Smart clothing to alleviate low back pain		
		detection of Alzheimer’s disease in early stage		
optical coherence tomography (OCT)	near-IR is used to identify the fluid/biofilms build-up beyond the eardrum	noninvasive ear infection treatment and reduced dose of antibiotic usage with a precise imaging technique	machine learning algorithm to detect middle ear fluid/biofilm	^174^
bionic man	system-based control of the brain (neuroprostheses)	paralyzed patients can interact with their environment (under development)	regeneration and replacement of the repaired organ/tissue with modern sensing and biomaterials	^175^

**Table 2:** Methods of Dynamic Hydrogel Development Strategies Adopted Along with Their Mechanism, Advantages, and Limitations

s.no.	fabrication method	principle/mechanism	advantages	limitations	ref
1	freeze–thaw cycling	repeated freezing and thawing of polymer solutions induces physical cross-linking via crystallite formation	no toxic cross-linkers; good mechanical stability; simple process	limited control over pore size; long processing time	^78,79^
2	ionic cross-linking	divalent or multivalent cations cross-link ionic polymers (e.g., calcium ions with alginate)	mild conditions; suitable for cell encapsulation	weak mechanical strength; ion exchange may cause instability	^86^
3	photo-cross-linking	light-activated photoinitiators generate radicals to covalently link polymer chains	precise spatial control; tunable mechanical properties	requires photoinitiators; UV may damage cells	^86^
4	enzymatic cross-linking	enzymes catalyze covalent bond formation between polymer functional groups	biocompatible; highly specific reactions	slower reaction rate; cost of enzymes	^80,83^
5 ^86^	click chemistry	bio-orthogonal reactions (e.g., thiol–ene, azide–alkyne) form covalent linkages	fast, efficient, high yield; cytocompatible	requires chemical modification of polymers	^78–80^
6	3D bioprinting	layer-by-layer deposition of bioink to create 3D constructs	custom geometries; integrates cells during fabrication	requires optimized bioink rheology; printing speed limitations	^87^
7	microfluidics	flow-focusing or droplet microfluidics to generate uniform hydrogel microspheres	high control over size; scalable	complex setup; limited to small-scale production	^80,83,88^
8	electrospinning-hydrogel hybrid	integration of electrospun nanofibers into hydrogel networks	improved mechanical properties; ECM mimicry	multistep fabrication; potential solvent toxicity	^89^

Hydrogel science and engineering advancements are possibilities in various disciplines, improving treatment and diagnostic methods.^90^ Hydrogels, which are characterized by a high water content and good biocompatibility, are becoming popular as scaffolds in tissue engineering applications in regenerative medicine.^91^ Hydrogels, which are created to mimic the extracellular matrix (ECM) for cell growth and tissue engineering, including wound healing and cartilage regeneration, are some of these innovations.^92^ Furthermore, self-healing hydrogels, whose characteristics change in response to environmental stimuli, are being fabricated for some innovative drug delivery systems where therapeutics can be released in response to certain circumstances.^93^ Hydrogels have applications in diagnostics, where biosensors are designed to detect biomarkers via hydrogels with high sensitivity for early disease diagnosis. For example, hydrogel-based platforms enable improvements in glucose monitoring and cancer biomarker detection, as they have become structurally stable and sensitive to various environments.^94^ Overall, these developments highlight the potential of hydrogels in enhancing the field of medicine and individualized approaches to treatment.

### 2.6 Bioprinting Based on Their Atomic Scale: 3D–4D Bioprinting Applications

3D bioprinting is an enhanced application of “additive manufacturing” involving layer-by-layer tissue or organ construction via a bottom-up technique.^105^ The objective of 3D bioprinting is to restore the typical structure and operation of complex tissues by depositing materials and cells in a specific pattern that mimics the natural cellular architecture.^106^ Cells or biomolecules are programmed directly onto a surface via 3D bioprinting to adhere together to build the required 3D construct.^65,107^ Cell adhesion, growth, and conformational changes can be influenced by ‘nanoscale’ characteristics, including the roughness and channels of bioprinted materials.^108^ The impact and aspects of the dimensional influence are shown in Figure 5.

**Figure 5:**
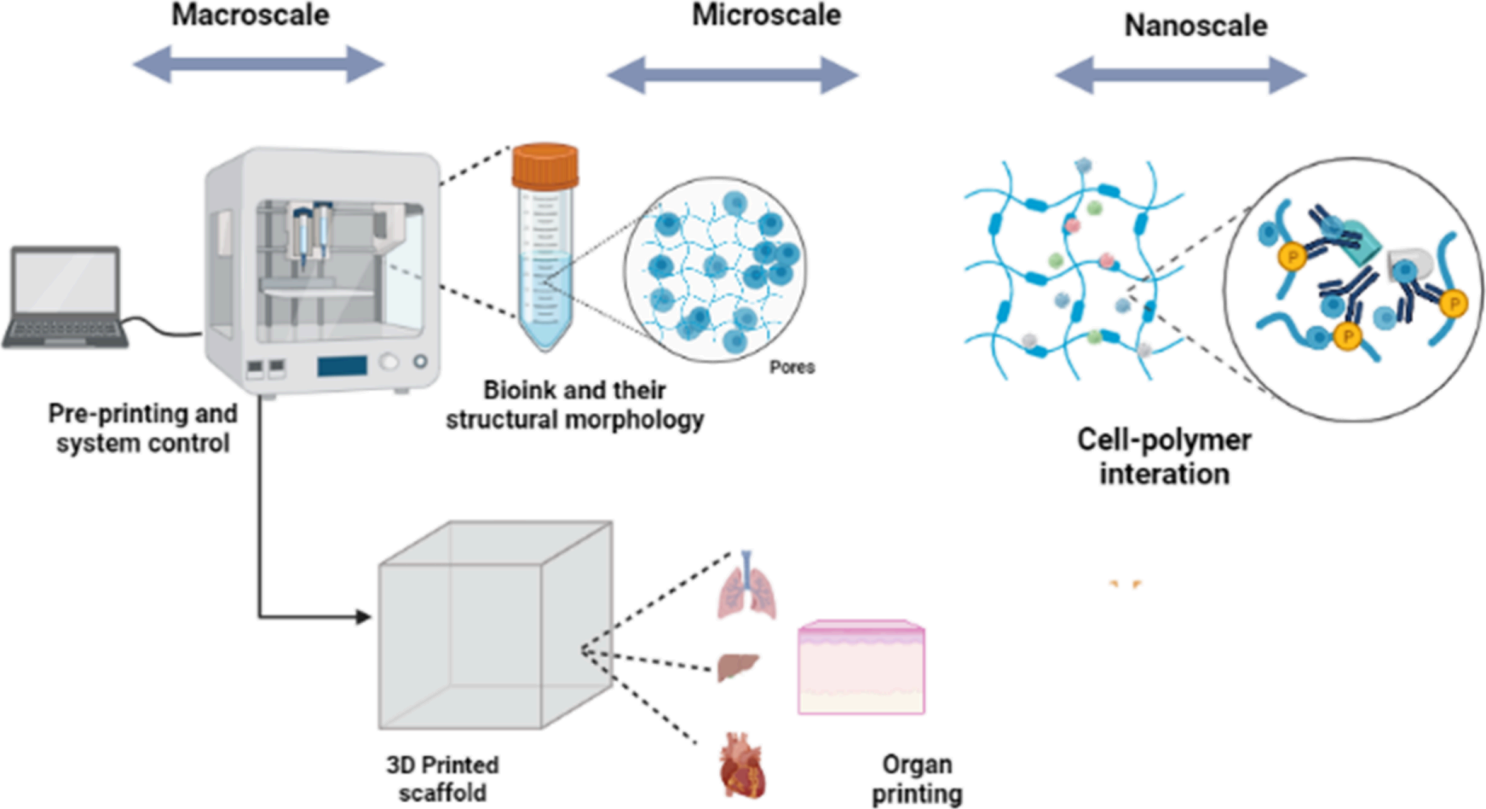
Impact of the dimensional variation of bioprinting in different applications. In the macroscale dimension range, the system controls the bioink formulation; in the microscale dimension range, the pores and structural morphology are viewed; and in the nanoscale dimension range, the seeded cells and polymeric interactions are observed.

Bioprinting involves in vitro seeding of the cells and tissues of interest. Therefore, it considers the modalities associated with living tissues, such as material biocompatibility, cell sensitivity to printing processes, adaptation of growth factors, and perfusion in the biomaterial fabricated.^109^ Because the entire procedure is programmed, consistent cell patterning and an ECM architecture can be achieved. The interconnected pores in bioprinted tissues are optimal for gas and nutrient perfusion and inter- and intracellular communications because of their layer-by-layer design.^110,111^ One of the significant steps is to select “bioink” for bioprinting processes carefully.

bioink is a polymeric substance of a hydrogel with a mixture of hydrogels laden with cells uniformly spread over a substrate to achieve the desired output.^112^ Different strategies are employed in bioprinting processes such as stereolithography (SLA), inkjet printing, extrusion-based bioprinting, and laser-assisted bioprinting (LAB). When the bioink laden with cells passes through the photo source on the substrate in stereolithography-based bioprinting, the photo source aids in solidification. In inkjet bioprinting, the fusion deposition method, which is based on bioprinting, is carried out in a drop-by-drop manner, laying down a bio-link to achieve layer-by-layer deposition through an external force (heat or pressure) on the surface of the substrate. The design control system is associated with the software.^87^ The input file used to design the design object is programmed, and standard triangle language (STL) files are spliced into G-codes, which are utilized for bioprinting.^113^ The overall design and instrument operation are programmable, and the essential properties must be considered. The bioink is printed through the micronozzle upon applying pneumatic pressure through the plunger or syringe. Moreover, printing depends on the sterility, nozzle diameter, and pressure applied to the cells, which affect their morphology.^114^ These factors influence the design and structure of the bioprinted scaffolds. Furthermore, the field has diverse clinical applications.

The bioink formulation considers the viscosity, stability, and strength for adapting cells to grow. The bioink formulation utilizes polymeric substances from natural, synthetic, and decellularized bioinks.^115^ Additionally, they are hybridized with two or more components via cross-linkers to achieve the expected physical and mechanical strength range, which is preferable for the bioprinting strategy employed.^30,116^ Cellulose, alginate, silk fibroin, collagen, gelatin, gelatin methacrylate (GelMA), hyaluronic acid, and chitosan are natural polymers in natural polymer-based bioinks.^117,118^ Natural polymers have greater advantages in terms of biodegradation and a greater chance of biomimicry in ECM matrices.

Additionally, they decreased cytotoxicity to the microenvironment of the human host system. PCL, PVA, PEG, and nanosilica-based bioinks are also hybridized with natural polymeric blends for increased strength to accommodate cells.^12,119^ Figure 6 illustrates the bioink formulation and printed matrix application in therapeutics. From the perspective of neuronal origin, a 3D, bioprinted organ is functionalized and used for implants to replace the repaired or damaged host organ.^120^ 3D bioprinting through 3D skin grafts has been a great tool for treating burns and autoimmune disorders. Induced angiogenesis is observed in 3D-printed scaffolds incorporated with platelet-rich plasma, but it does not cause fibrosis. This is achieved by implanting naturally occurring platelet-rich plasma (PRP), which autologously releases growth factors such as vascular endothelial growth factor (VEGF) into a 3D printable hydrogel scaffold.^120–122^ A research team developed a biomaterial for treating cardiac diseases by developing an injectable hydrogel. The hydrogel was introduced and characterized for its pore-forming ability, which helps resist the increased frequency of sound.^60^ The new venture in food technology is the development of artificial meat to avoid butchering the lives of animals. Mature bovine cells and stem cells are introduced into the hydrogel and grow into mature fat and muscle, resembling artificial meat in terms of flavor, smell, and taste, by MedTech 3D, an Israeli firm.^123^

**Figure 6:**
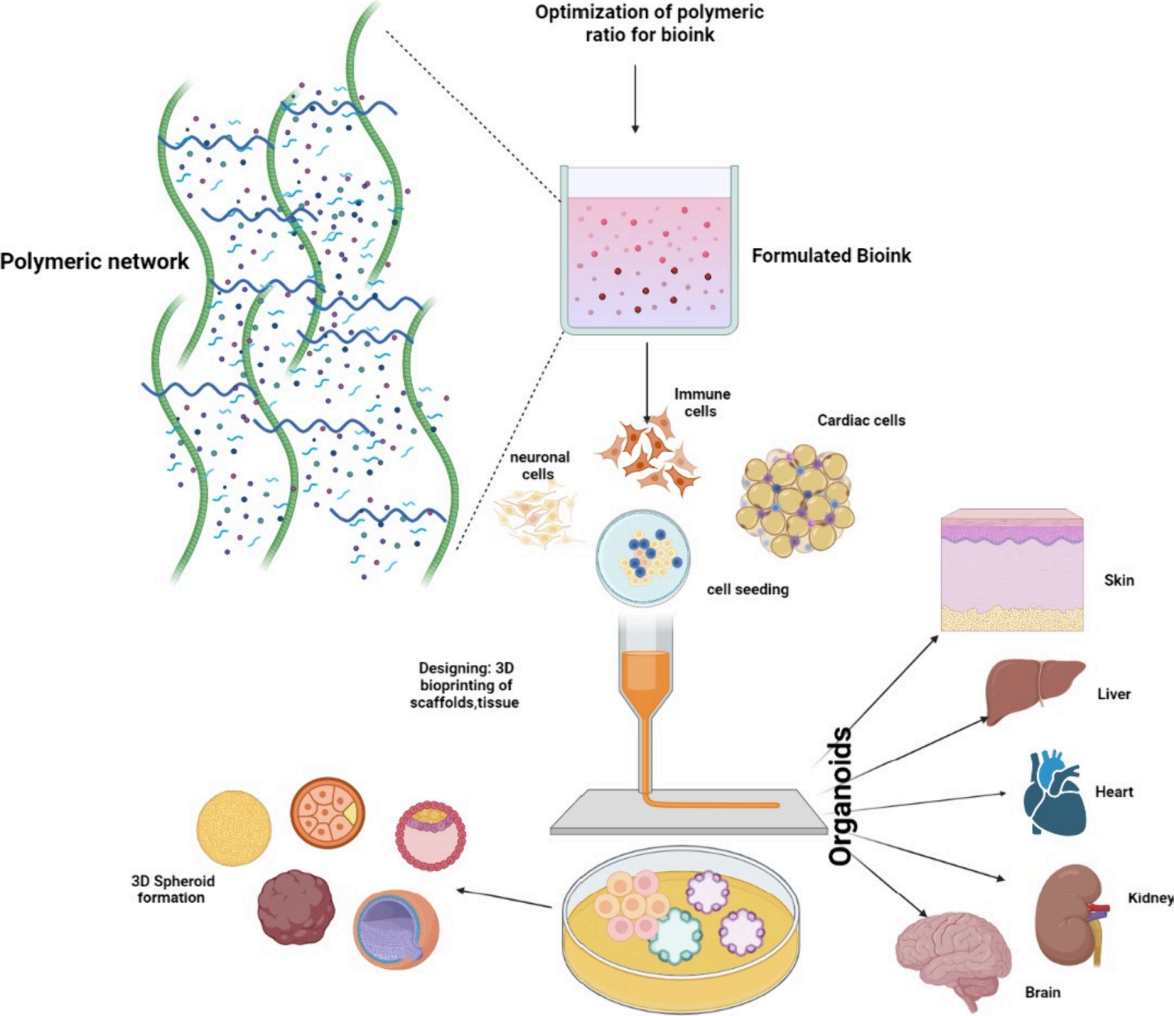
3D bioprinting: A step-by-step process of bioink optimization, bioink components, biomaterial design, and application for developing 3D bioprinted scaffolds for organ printing. A solution for new organ development.

In drug delivery, microbial bioinks have recently broken contamination barriers and have been successfully used in the drug delivery and 3D printing of living materials. *E. coli* was cultured, and the modified nanofibers were placed into the ink. The alginate mixture and the bacterial culture were printed on the calcium chloride surface as a cross-linking strategy.^116,124^ The controlled release qualities of the 3D-printed tablets decreased the incidence and number of dosages consumed by patients in their daily routine. For patients who take medicine numerous times a day, a reduced dose frequency extends the release of products and improves patient compliance.^125^ A 3D-printed composite scaffold with antibacterial effectiveness was developed to treat bone infections following craniotomy. Antibiotic-loaded PCL/hydroxyapatite structures enclosing macrophages in a hydroxyapatite-based hydrogel were printed. The composite scaffolds were implanted in a bone defect model via biofilm-associated *Staphylococcus aureus* craniotomy.^107,126,127^ Macrophages may release cytokines and chemokines that augment the antibacterial activity of other glia and leukocytes involved in infection, promoting biofilm dispersal. Following antibiotic treatment, the bacteria changed to a metabolically sensitive type. These findings suggest that treating established biofilms could be a viable alternative to antibiotic therapy.^128,129^ Through drug loading in biodegradable polymers such as polycaprolactone (PCL)/poly­(lactic-*co*-glycolic acid) (PLGA), sustained drug release can be achieved via 3D printing technology. They also contribute to cell delivery systems, pulsatile and aerosolized systems, and high-throughput drug screening, significantly contributing to coronavirus disease (COVID-19) treatment strategies due to their precision-based delivery mechanisms.^128–131^

The advancement of bioprinting has improved the clinical use of this technique and opened up opportunities in regenerative medicine, tissue engineering, and personalized medicine.^132^ Tissue engineering involves depositing layers of bioinks to form multicompartmented tissue constructs, which addresses problems associated with organ transplantation and regeneration.^133^ In particular, synthesizing bioinks based on hydrogels and living cells has facilitated the fabrication of physically and mechanically functional tissue constructs. For example, current research has shown the possibility of printing blood vessels and cardiac tissues essential in tissue repair and regeneration.^134^ These developments are especially crucial for the topic under analysis because the cells built by the bioprinter may help to minimize the need for donor organs. In dermatology, bioprinting has created skin grafts that help with faster tissue healing and integration.^135^ There has been a development in printing multilayered skin models with better structural and functional properties suitable for chronic wounds and burns.^136^ In addition, bioprinting is taking the field of personalized medicine a step further by allowing for the printing of tissue constructs according to the patient’s specifications. For example, implants and prosthetics customized to the patient’s needs with respect to their anatomy and physiology may be printed to increase operative efficacy and patient satisfaction.^137^ The introduction of bioprinting technologies to the clinical setting could usher in a new era of tissue engineering and regenerative medicine, providing tailored and scalable solutions for several diseases.

In 3D bioprinting, the printability of a hydrogel is determined by its gelation kinetics, shear-thinning behavior, and yield stress. Gelation kinetics describe the rate at which the hydrogel transitions from a liquid to a solid-like state.^85^ A slow gelation rate can result in filament spreading or structural collapse, while overly rapid gelation can cause nozzle clogging and irregular extrusion. Shear-thinning behavior, where viscosity decreases under applied shear stress, facilitates smooth extrusion through the nozzle and allows the material to rapidly recover viscosity after deposition, thereby maintaining shape fidelity.^86^ Yield stress is the minimum stress required to initiate flow; hydrogels with an adequate yield stress can resist deformation and prevent printed layers from sagging under their own weight.^113^

A key challenge lies in reconciling the mechanical requirements for structural stability with the biological requirements for high cell viability.^138^ Hydrogels with high stiffness or dense cross-linking provide print resolution and structural support but can impair nutrient diffusion and impose mechanical stress on encapsulated cells.^80^ Conversely, low-stiffness hydrogels are more permissive for cell survival and proliferation but often lack the structural integrity to maintain complex geometries. To address this balance, strategies such as composite bioinks combining a mechanically robust, printable polymer with a biologically favorable gelling polymer or dual-stage cross-linking systems are employed.^139^ In the latter, an initial mild gelation step preserves cell viability and shape fidelity, followed by a secondary cross-linking stage to enhance long-term mechanical stability.^140^ These approaches enable fine-tuning of the hydrogel properties to achieve both high printability and optimal biological performance.

By enabling shape memory characteristics and responsiveness to external stimuli, 4D bioprinting adds a functional dimension, time, to 3D bioprinting. This method enables printed scaffolds or structures to experience predetermined, reversible deformations in response to particular environmental stimuli, including light, magnetic fields, pH, humidity, and temperature.^140^ By adding intelligent biomaterials to the printing process, the construct gains “intelligence” that allows it to change its shape, mechanical characteristics, or purpose after it has been manufactured. For instance, bioinks based on shape memory polymers have been developed to mimic dynamic tissue morphologies found in nature by folding or unfolding in response to temperature changes.^141^ In order to allow lumen opening or closure based on physiological conditions, temperature-responsive hydrogels, such as poly­(*N*-isopropylacrylamide) (PNIPAAm), have been integrated into 4D-printed vascular scaffolds. The ability of the printed scaffolds to deform in a programmed way in response to particular stimuli is made possible by the fourth dimension of 4D printing, which opens up new possibilities for functionalized organ or intelligent biomaterials.^142^ Smart hydrogel-based inks have been developed recently to enable the development of programmable deformations in 4D-printed scaffolds. Drug delivery systems have used pH-responsive hydrogels with chitosan or poly­(acrylic acid) to achieve controlled release in various tissue microenvironments. Hydrogel scaffolds embedded with magnetic NPs have the ability to change shape or orientation when exposed to an external magnetic field, which may have uses in remote-controlled tissue engineering.^143^ When exposed to particular wavelengths, light-responsive hydrogels containing azobenzene derivatives or spiropyran moieties have been used to cause localized deformation or drug release. These illustrations show how 4D printing broadens the range of applications for conventional 3D scaffolds, creating opportunities for intelligent biomaterials that can self-adjust, self-assemble, or perform adaptively in intricate biological settings.^144^ Figure 7 illustrates the modification of the printed scaffold based on the external stimuli applied over the scaffold.

**Figure 7:**
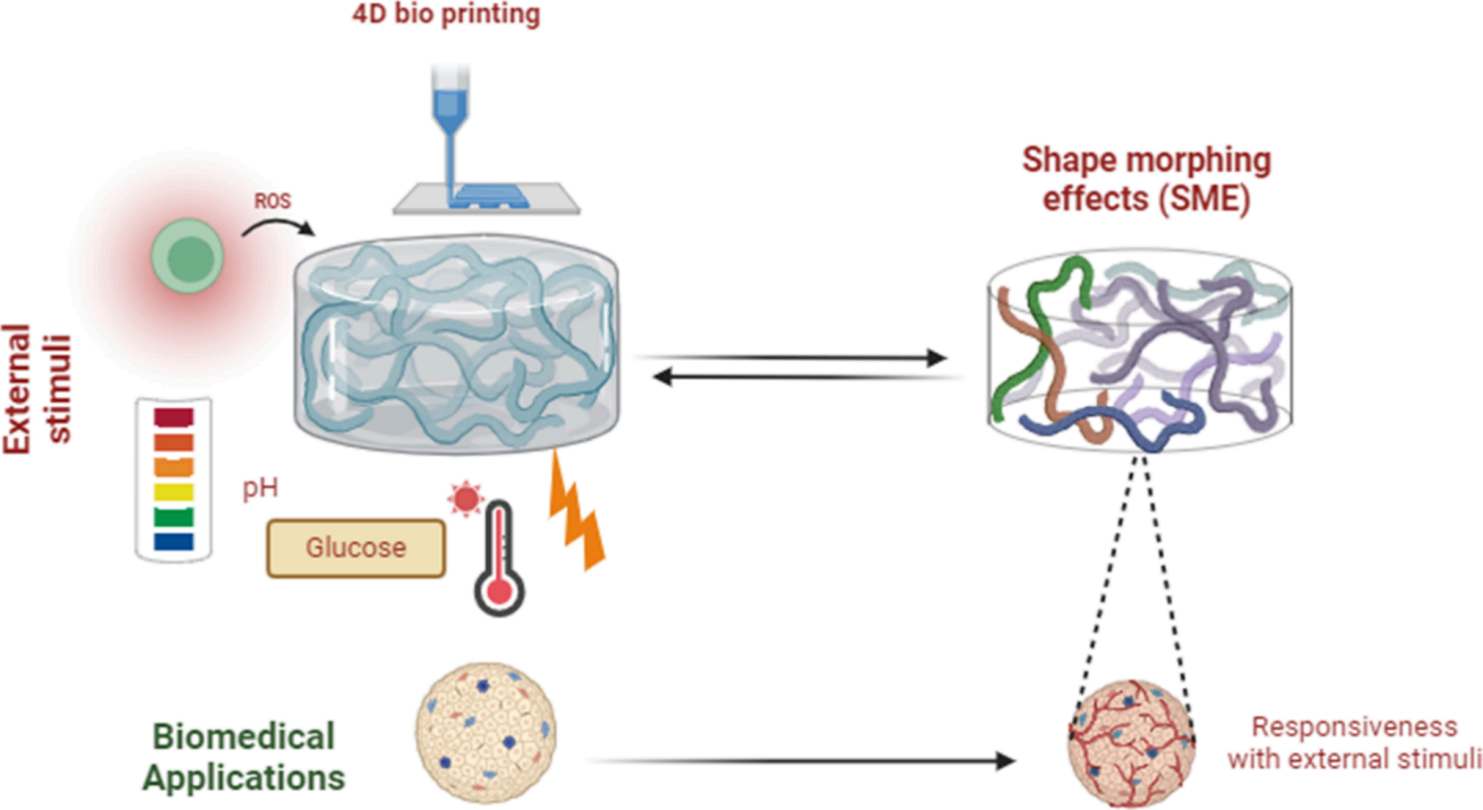
Shape memory effects incorporated in biomaterials used for 4-dimensional printing and their ability to respond to external stimuli.

### 2.7 Novel Applications of Nanofibers

The fibers with dimensions of less than one nm are nanofibers. They are fabricated from various polymers and thus have several physical properties and potential applications. Owing to their greater surface area and porous nature, mimicking the ECM promotes better cell–matrix interactions and cell infiltration into the scaffolds. Nanofibers can be prepared via dry spinning, electrospinning, or wet spinning processes. Electrospinning is a commonly adopted method because of its ease of use and better control over fiber characteristics. This approach uses electrostatic forces to produce nanofibers with a greater surface area and excellent pores. This approach uses electrostatic forces to produce nanofibers or particles with relatively large surface areas and perfect pores. Needle-based and needleless electrospinning systems are two methods employed in nanofiber fabrication.

Bioactive molecule-loaded electrospun nanofibers use PCL for skin regeneration and wound healing treatments.^95^ The controlled delivery of multiple drugs is made possible via the electrohydrodynamic (EHD) technique, which has potential applications in cancer treatment strategies.^96^ Bioactive molecules such as growth factors, drugs, and DNA are incorporated in the coaxial process to deliver genes, growth factors, and other targeted delivery mechanisms.^97^ Gelatin-grafted nanofiber mats (NFMs) are fabricated via polyethylene terephthalate (PET), and their surface is modified to mimic the ECM to accommodate endothelial cells.^98^ The gelatin grafting matrix with more carboxyl groups throughout the matrix has the potential to spread and increase the number of endothelial cells by preserving their phenotypes.^31,99^

A biodegradable poly­(L-lactic acid) porous scaffold fabricated via an electrospinning technique is seeded with nerve stem cells (NSCs).^96^ The improved ability of NSCs to adhere to the platform supports neurite outgrowth for neural tissue engineering as potential support to restore the central nervous system (CNS) via delivery of therapeutic molecules and growth factors.^96,100^ This technique supported the development of a biodegradable nanofiber-based face mask with air filtration and antimicrobial capabilities. Cellulose, chitin, keratin, chitosan, PVA, PLA, and polypropylene (PP) are a few of the polymers used for designing face masks that fulfill the criteria required for the design of face masks.^101^ The blends of two or more polymers are also used to prepare antimicrobial fibers. The critical parameters in electrospinning and its applications are listed in Figure 8.

**Figure 8:**
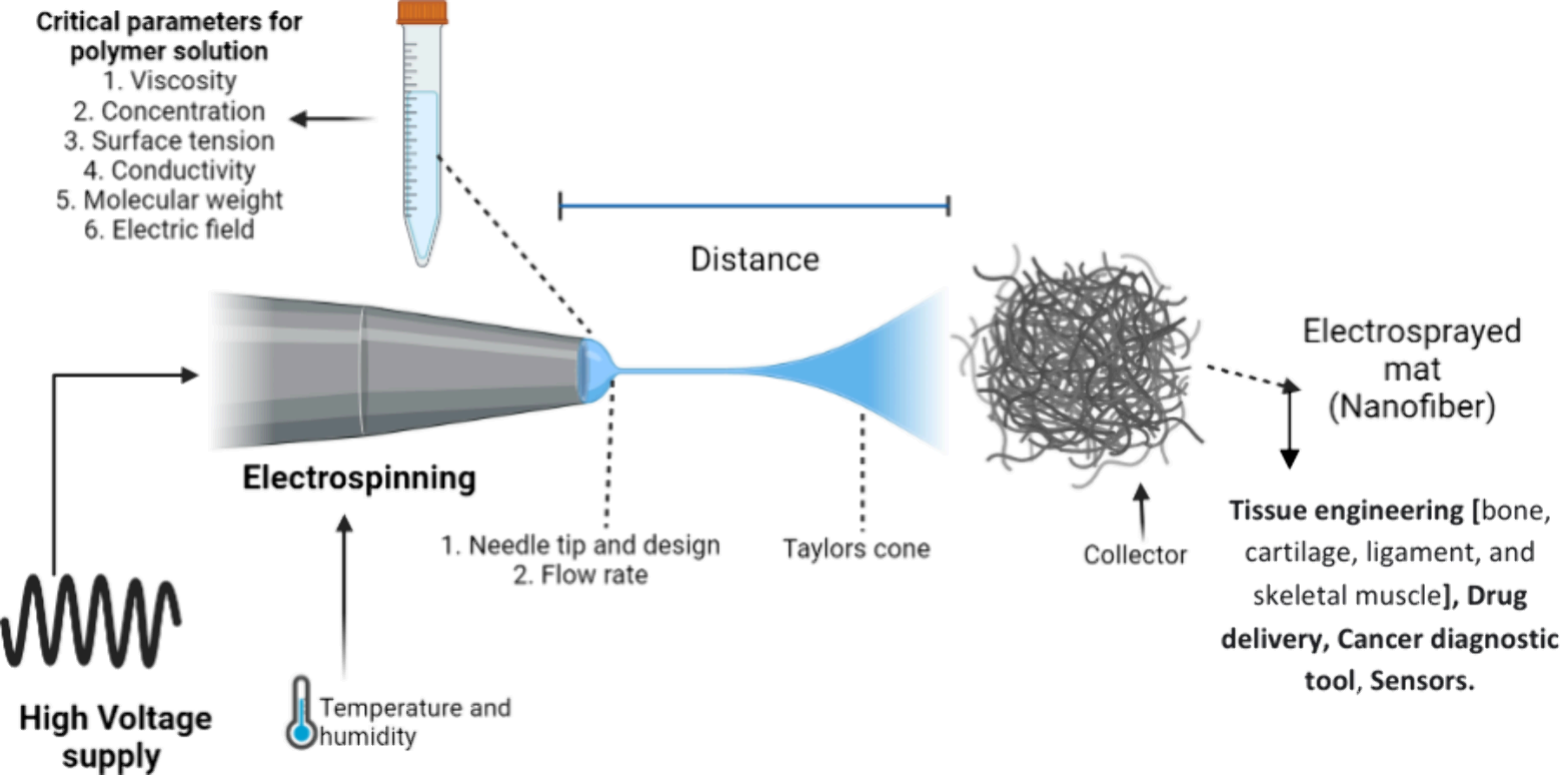
Critical parameters of electrospinning and the formation of nanofiber mats on a substrate with end-use applications in tissue engineering, drug delivery, diagnosis, and sensor applications.

Therefore, advances in nanofiber technology have led to a broad range of clinical uses. The nanofiber electrospun originates from materials forming a scaffold, enabling regenerative medicine of nerves, bones, and skin.^102^ These nanofiber scaffolds allow for cell deposition and dispersal, which result in efficient tissue regeneration. Nanofibers are also used as therapeutic delivery systems for site-specific and controlled release of drugs and other therapeutic agents with enhanced bioavailability and reduced side effects, especially in cancer and chronic disease therapy.^103^ In diagnostics, nanofiber-based biosensors capitalize on a high surface area and functionalization for biomarker detection to help diagnose early disease, including cancer or cardiovascular diseases.^104^ Furthermore, nanofibers are revolutionizing molecular medicine via novel tissue engineering, smart drug delivery, and personalized diagnostics.

### 2.8 Microfluidics

Microfluidics is a broad range of applications in which the system’s processor manipulates fluids precisely in microscale dimensions via multiple channels.^145^ The volume of the liquid used is in the range 10^–9^–10^–18^ L. Therefore, the volumetric forces are greater than the surface forces. These significant features show that reduced weight, high throughput, and volume have made the device portable and efficient for practical analysis.^146,147^ A microfluidic chip is a glass or polymer with molded or engraved microchannels. Several holes of various dimensions carved out through the chip connect the microchannels built into the microfluidic chip to the macroenvironment. Fluids are injected into and expelled from the microfluidic chip through these routes. Fluids are directed, mixed, separated, or manipulated to achieve multiplexing, automation, and high-throughput systems. The microchannel network design must be meticulously detailed to accomplish the necessary qualities.^148^ They have a significant application in detecting pathogen electrophoresis of proteins and nucleic acids—the analysis of DNA and lab-on-chip models. The design and selection of materials are sensitive to radiation and other aspects of characterization. The usual material of polydimethylsiloxane (PDMS) or poly­(methyl methacrylate) (PMMA) is among the most preferred materials for producing substrates and sealing layers. However, they are sensitive to X-ray and UV radiation.^145,149^ Figure 9 provides an illustration of microchannels in microfluidics that have diverse applications in high-throughput screening and lab-on-chip models.

**Figure 9:**
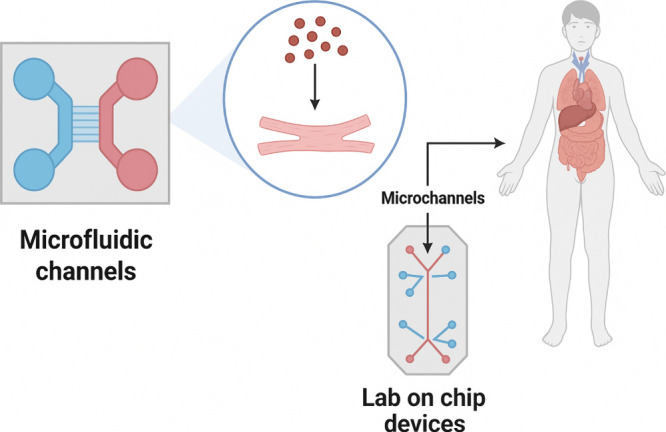
Microchannels in microfluidics have rapid or diverse applications for high-throughput screening, detection, and analysis, with end-use applications such as lab-on-chip models and their clinical applications in rapid diagnosis.

The microfluidic chip was prepared by cutting a square-shaped glass chip (3 × 3 dimensions) and sterilizing it with piranha solution at 60 °C. In addition to a channel reservoir, microchannels (C1, C2, C3, and C4) are prepared via photolithography in sterile room conditions and utilized as a substrate.^75^ The microchannels were treated with 60 nM chromium before the chemical etching process to obtain smooth tracks. After glass corrosion, three electrodes were created via a second photolithography mask, and gold was deposited on the channels via magnetron sputtering. The three gold electrodes were deposited later, and the device was drilled to connect the inlet and outlet tubes. In a step-by-step process, the sealing step involves an adhesive, a thin film composed of Norland Optical Adhesive and polyester. This was followed by curing at 50 °C for 12 h to improve adhesion. The polyester film is successfully cut from the sealed device, and the glass is supported by PDMS to increase the physical strength.^145^

Microfluidics aids in the detection of pathogens or cells of interest in diagnostic and cell sorting applications. Sensors, which are typically optical detectors, are interfaced with a microfluidic cartridge for visualizing or measuring the quantity of interest. Analytes can be defined, quantified, classified, and, in some cases, sorted via an optical detector. The optical detection system is possible with the criteria of a mature assay protocol (standardized with wavelength, fluorophores attached to the particles, and others) available.^146^ Optical detection in microfluidics has many applications, including biochemistry studies such as protein detection, cytometry, enzyme kinetics, cell biology, immunology assays, and screening and fluid manipulation. Absorbance, fluorescence, chemiluminescence, and surface plasmon resonance are the most commonly measured optical properties. Because of their ease of use, sensitivity, integration into the manufacturing process, and ability to alter chip optical properties, many device developers utilize such designs.^145–147,149,150^

A single slip chip was developed for DNA synthesis and scalable storage and was integrated into a DNA-based data storage system.^151^ Sequencing on the same electrode was achieved by incubating the electrodes in the polymerase solution in the fluidic channel and then exposing the electrode to four reservoirs holding different deoxynucleoside triphosphates (dNTPs). The electricity from the electrode was measured for sequencing purposes during the experiment. The overall accuracy of the chip was found to be 89.17%, and by error correction, it was able to reach 100%.^151^ This electrochemical DNA synthesis method helps manipulate data at any time, facilitating the ease of data storage in DNA, which can be helpful in various detection areas in the future.^152,153^ This platform has also benefited SARS-CoV-2 nucleocapsid protein identification via nasal samples via a hybrid of hydrodynamic microfluidic filtration and a sandwich immunoassay by specifically screening eight antibodies with increased affinity and specificity for SARS-CoV-2 via the on-chip procedure.^148^

Technological improvements in microfluidics in the past few years have implications in clinical practice by improving diagnosis, treatment, and drug targeting for specific patients. One of the emerging applications of microfluidic devices is in portable diagnostic systems that are used for the detection of infectious diseases such as COVID-19 or the tracking of various biomarkers from small sample volumes.^154^ One such advancement is the application of microfluidics for CTC and ctDNA isolation and detection to facilitate noninvasive diagnosis and cancer monitoring to tailor treatment options and evaluate treatment effectiveness in real time.^155^ Furthermore, in single-cell analysis, microfluidics has also played a significant role in the high-throughput cell profile necessary, especially concerning cell heterogeneity and cell-based therapy.^156^ In drug development, microfluidic platforms enable the screening of libraries of potential compounds that are likely to have effective therapeutic interactions, thus reducing the time to identify promising drug candidates while using a small amount of reagents and reducing the cost.^157^ In addition, intelligent microfluidic systems for genetic and molecular diagnostic assays and more advanced techniques for nucleic acid amplification and detection, including PCR and RT-PCR, have increased the sensitivity and speed of genetic testing.^158^ Organ-on-a-chip models, another important application, simulate human organ systems to identify the mechanisms of diseases and drug reactions for drug testing and disease modeling.^158^ Moreover, microfluidics is also revolutionizing blood diagnostics and hematology as it can analyze component parts of blood, such as its cells, cell count, and hemoglobin levels, faster and with enhanced efficiency. In conclusion, these developments indicate the role of microfluidics in revolutionizing clinical diagnostics, therapeutic methods, and personalized medicine.

## 3 Clinical Applications of Biomaterials

Biomaterials have end-use applications in the medical field to restore function and facilitate recovery from a wound or deformity. There are a few medical implants, wound healing scaffolds, diagnostic tools, biosensors, and drug delivery systems where biomaterials have value.^73^ The success rates of biomaterials or small molecules that have passed through clinical trials are lower. The significance of developing various biomaterials is scaling up to reach the clinical stage to ease medical intricacies.^11^ The design of a hydrogel for burn treatment involves the construction of a hydrogel dressing that automatically dissolves, creates an infection barrier, and promotes healing. The hydrogel allows on-demand dressing removal and re-exposure of the wound without mechanical debridement and cutting by dissolving into safe byproducts in a regulated manner, resulting in more accessible, less traumatic therapy.^159^ Blood vessel narrowing and complications are addressed with the development of dissolvable zinc stents. The developed bioabsorbable zinc stent corrodes harmlessly over time, alleviating the chronic hazards generally associated with permanent stents. Early trials with absorbable zinc stents have yielded positive results.^160^

Advances in biomaterials have also helped to cross or deliver drugs, surpassing the blood-brain barrier layer, to treat Alzheimer’s disease, Parkinson’s disease, and brain tumors. Advanced ultrasonic techniques are utilized to pass through the BBB momentarily and safely, allowing drugs to target brain tumors without the need for surgery. Biomaterials also play a vital role in enhancing the targeted intracellular delivery of drugs, as each cell includes intrinsic systems that recognize and eliminate potentially dangerous compounds and foreign objects.^19^ As a technique for delivering medicines to specific cells, more engineering is needed to ensure that the treatments reach precise structures within the cells. Intelligent delivery systems can evade cellular defenses, transport pharmaceuticals to selected intracellular areas, and release drugs in response to specific molecular signals for future healthcare treatments.^161^ Figure 10 illustrates the feedback loop of innovation of the biomaterial from the bench to the bedside.

**Figure 10:**
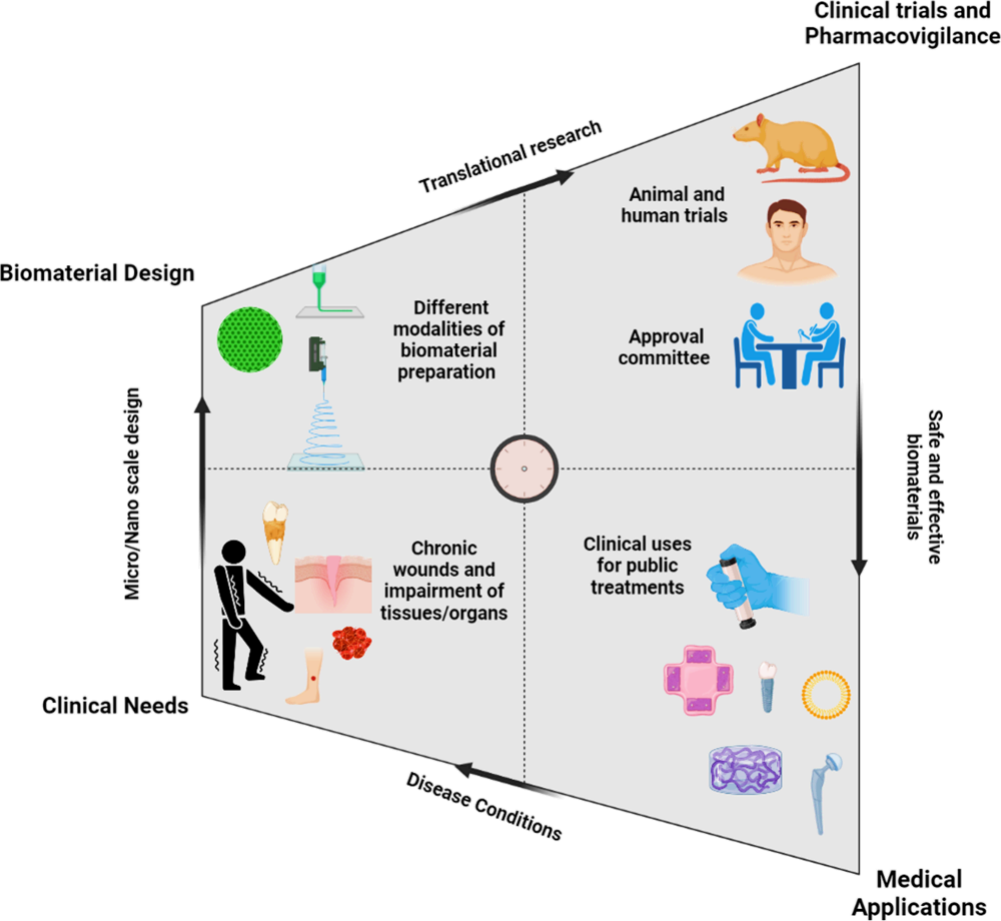
From biomaterial design to clinical translation: A feedback loop of innovation. Steps involved in translating the biomaterial, different approaches used, and the need to meet the clinical demands for a biomaterial to reach clinical uses.

Microneedle-based drug delivery has paved the way for delivering drugs noninvasively to the targeted site and route of therapy. Dissolvable microneedles, which can be disposed of safely, are being designed for vaccination.^162^ Nano vaccines with porous silica rods on the surface of the 3D scaffold are administered for cancer treatment to delay tumor growth by manipulating immune cells for a robust immune response. Nanovaccines are combined with bacterial DNA to trigger an immune response by forming a flower-like complex that instructs foreign cancer cells to trigger an immune response.^163^ Nuclear medicine is also used to detect and treat several microbial infections by providing a precise imaging tool for diagnosing diseases. The biophysical aspects of COVID-19 treatment strategies help inhibit SARS-CoV-2 replication by targeting interactions between the nucleocapsid (N) protein and its binding partners, which could be a viable option.^164^ Furthermore, the applications of biomaterials are rising to the standard with advances in computerized detection and treatment strategies. In Table 1, several significant applications and characteristics are mentioned.

The COVID-19 outbreak began in December 2019, and in early 2020, it triggered massive disruptions in the global economic and health sectors. However, its impact on biomaterials and the tissue engineering market is relatively short-lived. The COVID-19 pandemic has prompted new developments in various materials and technologies, especially those used for diagnosing and addressing COVID-19.^176^ Tissue engineering is the process of developing biomaterials, scaffolds, and regenerative techniques and is a USD 125 billion global industry. The row is expected to grow to USD 144 in the near future. In 2022, the Engineering Procurement and Construction (EPC) sector reached USD 78 billion and may increase to USD 299.^176^ This growth is equivalent to a compound annual growth rate (CAGR) of 15%. Last, metallic biomaterials, including titanium, stainless steel, and cobalt–chromium alloys, exhibited high market demand from 2019 to 2022. The growth rate in this segment primarily arises from the increase in the number of geriatric patients who require stronger and more biocompatible implants or prosthetic devices. Similarly, the growth in the tissue regeneration and wound healing sectors also fuels this growth with the help of increasing healthcare investments and a growing patient population. Additionally, the worldwide 3D bioprinting market reached USD 869 million in 2019 and is expected to experience continuous growth in the coming years.^177^ The global biomaterials market was valued at approximately USD 140 billion in 2023. It is projected to reach approximately USD 160 billion by the end of 2024, reflecting a robust CAGR of approximately 12–14%.

## 4 Next Generation of Biomaterials

Understanding how interfaces interact with biomolecules is critical for developing intelligent and responsive biomaterials for healthcare, industrial, and nutritional-based applications.^178^ The processes occurring at the surfaces of artificial components and organisms are governed by chemical and nanotopography.^6,179^ Monitoring such communication would enable materials to perform a variety of new generation materials, such as biomedical implants with programmable behavior, bioengineered scaffolds that can support the growth of specialized cell types, and biosensor surfaces that can efficiently endure protein and microbial fouling.^19^ The development of a surface that resists fouling and degradation in the surface range can be designed to maintain the stability of medical implants inside the host microenvironment, which will lead to the development of next-generation biomaterials.^179^

The complexity of highly efficient biomaterials influences their translation to clinical use. A simple biomaterial design can help the material pass through regulatory checkpoints. This complexity creates a need to focus on the design parameters of biomaterials with the minimum level of complexity for engineering clinical applications.^180^ Recently, complex technologies incorporating next-generation bioassays and high-throughput screening have simplified cell–biomaterial interactions.^28^ The practical and regulatory limits imposed on the synergy of newer technologies for biomaterials integrating biologics, medicines, or cells should be considered when the plethora of materials currently under study is translated.^181^ The design parameters combine physical and chemical aspects for better biological functionality and applications. In the physical part, a uniform networking pattern, viscoelasticity, and pore size distribution have been built into biomaterials via a variety of processes.^182^

In contrast, self-assembly and molecular recognition breakthroughs have enabled the fabrication of many structures, such as fibers, tubes, and rods. From a chemical perspective, the binding of a drug with a polymer contributes to the enhanced mechanism of the material’s action in therapeutics.^183^ The design parameter also includes a factorial design for the accurate optimization of parameters. The modeling consists of partial and complete factorial designs for screening and surface response evaluation.^184^ The implantable biomaterial is further optimized via in vivo experiments and functional assays to reach the clinical stage.^185^ Proteomics plays a pivotal role in sequencing and high-throughput evaluation in biological assessments to evaluate growth, proliferation, and morphology. Furthermore, the patterning of the biomaterial is possible via the automatic design of features via 3D printing technology to achieve a defined architecture of the biomaterial.^2,146,185,186^ A few significant factors to be considered for biomaterial design are as follows: 1. Cytotoxicity, 2. Biocompatibility and degradability, 3. Cell viability, 4. Structural stability. Figure 11 illustrates the next-generation biomaterial features, biomedical applications, and smart biomaterials.

**Figure 11:**
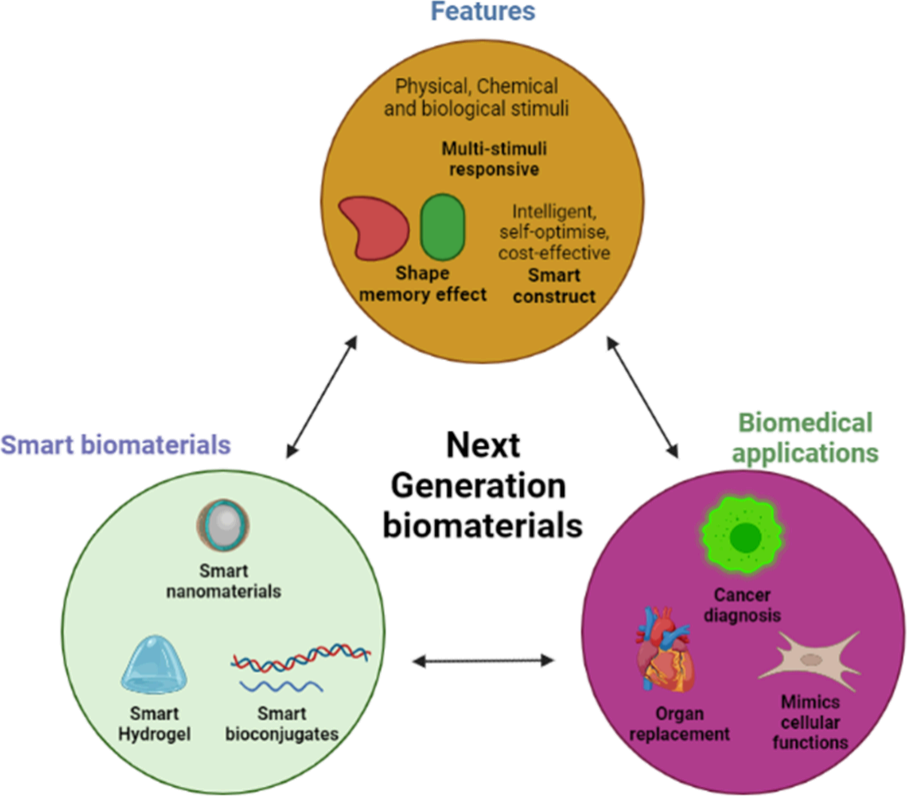
Next-generation biomaterials with smart constructs and distinct features for future applications in the biomedical field for diagnosis, treatment, and drug delivery applications.

The dimensional range of nanoscale and microscale architectures of biomaterials, from network orientation in 3D materials to nanotopography and surface properties, has a powerful additive effect on the strength of the material.^11^ Material simulation techniques exist for each dimension and can be combined to capture mechanical behavior, transport characteristics, degradation, and collapse.^184^ Biomaterials have evolved in three generations and have aided in bone regeneration, tissue regeneration, and signaling.^26,106,187^ The molecular impulses between cells and their surrounding matrix are dynamic across a wide range of time and dimensional (nm–cm) scales, and the biophysical properties of tissues also influence them.^188^ Cellular pathways are governed by many factors, including sticky interstitial contacts, cytoskeletal and ECM remodeling, and agonist and antagonist gradients.^115,183,189^

The sterility and safety of the biomaterials and the data that shed light on the probability that using the medical device can harm the patient are necessary to conduct a risk evaluation of any medical device. The use of nontoxic or low-toxicity chemical components, including biomaterials, is one method to achieve this goal.^144^ To assess the possible toxicity of a biomaterial or medical device, the ISO 10993 series is used for the rapid screening of medical equipment. The animal species used may respond to medical equipment differently from how humans do. However, in most cases, combining in vitro and in vivo testing can provide adequate information to assess a medical device’s possible toxicity, which is crucial for the evaluation of risks and safety.^144^

### 4.1 Biomaterial Design Using AI and ML Tools

AI technology has revolutionized the discovery and advancement of biomaterials and drugs, contributing to improvements in processing power, extensive data analysis, deep learning, and simulation modeling.^16^ Although ML and its techniques have a wide range of applications outside data science, researchers who specialize in biomaterials have restricted their applications to biomaterials. High-level computing tools can accelerate the development of biomaterials while eliminating the need for conventional trial-and-error techniques. Image analysis is a crucial activity for which ML has proven beneficial.^190^ Consequently, object identification and image classification techniques can be applied to automatically recognize and pinpoint the existence of specific items in an image or video. The algorithms learn to identify informative areas in the image and extract features such as edges or particular forms from them by being fed example images as input.^191^ Currently, similar methods are widely used for tasks such as facial recognition and autonomous driving. However, this methodology has enormous potential for use in other situations where decisions are made on the basis of visual impressions.^192^ Cell nuclei were successfully identified via microscopic analysis; microtissue-contraction analyses were automatically performed in scientific experiments, and 3D printing processes of biomaterials were improved. Other achievements include successfully extracting the steady progress of glaucoma, dementia, or cancer from medical images.^17^ Using the ML algorithm approach, it was feasible to investigate treatments focused on particular disorders. To investigate glycan functions, single-molecule sensing and production procedures, including 3D bioprinting or microparticle synthesis, should be increased.^193^ Therefore, next-generation biomaterials evolved to be assisted by robotics and were named to be an integration of biomaterials with proteomics, “Biomateriolomics.”^194^ Figure 12 explains the use of AI and ML tools in biomaterials and health care research.

**Figure 12:**
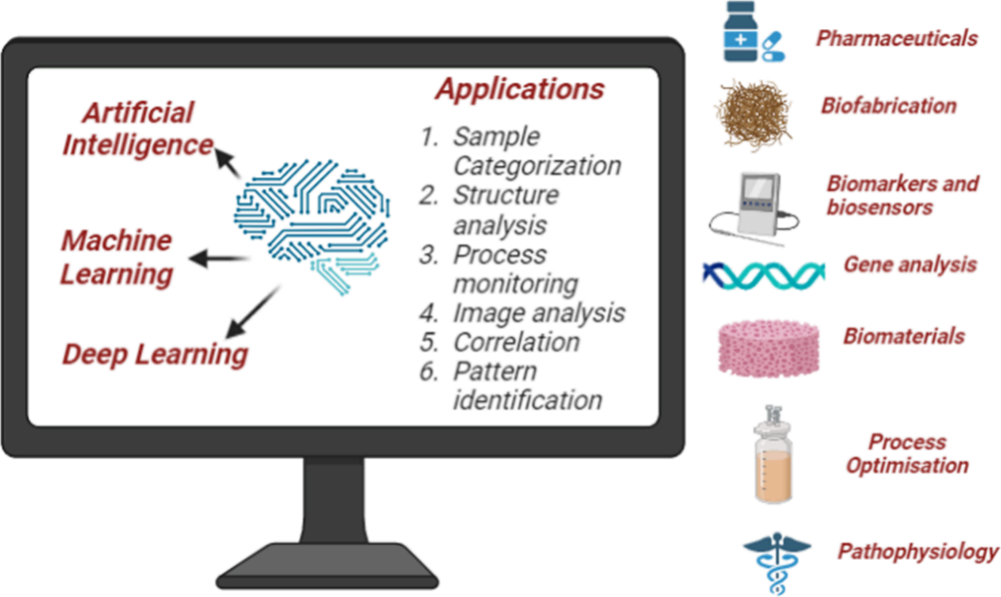
An overview of in silico approaches that help to induce intelligence in biomaterials, thereby leading to precision-based biomaterial design and diagnosis.

Biomaterial interfaces have therefore emerged as an innovative way to incorporate AI and ML in personalized medicine. Notably, with the help of AI, biomaterials that are optimized for specific biological environments of patients can be designed by analyzing large data sets.^195^ Current investigations have shown examples of how these computational methods can accurately predict material properties and biological effects, greatly expediting the identification and development of new biomaterials. For example, Kamal et al. reported in the perspective of 2023 that the application of ML could make it possible to predict the biocompatibility of polymeric materials that can be useful in tissue engineering, accelerating the screening of all polymeric materials.^196^ Specifically, the application of AI has been reported to improve the accuracy of different bioprinting approaches in 3D bioprinting to increase the degree of tissue regeneration. A significant goal in biomaterial research is to engineer entire tissues and organs that form functional tissue and organ grafts that can be assimilated with the host organism.^197^ Considerable breakthroughs have been achieved in this area through the improved knowledge of stem cell biology, 3D bioprinting, and biofabrication. Stem cells have been applied to fabricate patient-specific tissues when incorporated with advanced biomaterials. Their 2022 review reported that this is achievable by integrating stem cells with ECM-mimicking hydrogels to form bioengineered tissues with natural tissue behavior.^197^ In addition, advances in 3D bioprinting technologies, including multimaterial and cell-based bioprinting, have improved the ability to create intricate, composite tissues. Biofabrication strategies have also emerged and advanced, with recent advances concentrating on the fabrication of vascularized tissues needed for organogenesis.^83^ Biomaterials research has been revolutionized by recent developments in AI and ML, making it possible to predict structure–property-function relationships from sizable experimental and simulation data sets quickly. Today, polymers, composites, and hybrid materials with desired mechanical, thermal, or biological properties are designed by using deep learning and generative models. ML-driven multiobjective optimization speeds up discovery by balancing variables like mechanical stability, degradation rate, and biocompatibility. The creation of next-generation intelligent and sustainable biomaterials for tissue engineering, medication delivery, and medical devices is made possible by these methods, drastically reducing trial-and-error testing.^144^ These advancements suggest that introducing biomaterials with other advanced technologies in the near future holds promise for various organ transplantations and fully functional tissue replacement, thus filling the existing gaps in regenerative medicine treatment.

## 5 Conclusion and Future Perspective

In recent developments, the dimensions of biomaterials have been adjusted for better functionality, cell–material interactions, specificity, and biocompatibility. The synthesized biomaterials are translated for end-stage applications and are available for public use. Therefore, the development stage is evaluated through animal and human studies, and regulatory approval is received for clinical applications. Biological applications include tissue engineering, biomedicine, drug delivery, biosensors, diagnostic tools, medical implants, and bone and tissue grafts. The impact of the biomaterial design and strategies for designing biomaterials based on physical, chemical, and biological aspects is discussed.^198^ Moreover, recent technologies have been used to engineer signals in biomaterials by incorporating specific cues into design parameters. Computational modeling, design, and high-throughput screening of biomaterials help meet the checkpoints of regulatory evaluations.^140,199^ The evolution of biomaterials depends on their dimensions, synthesis techniques, and efficiency.^200^

The path of biomaterials takes up another level by connecting knowledge of disease conditions with the modification of biomaterials. This approach has made intelligent biomaterials possible. Intelligent biomedical technologies are emerging as the discipline of intelligent biomaterials evolves. The appropriate design of biomaterials has led to an increase in the size of the biomaterial market. Technological breakthroughs have eased the ability to repair tissue, bone, and organ deformities with a greater range of treatments. Although research on natural biomaterials is increasing, their clinical applications are relatively limited. Therefore, the global biomaterials market aims to find a solution to treat disease or deformation via intelligent technologies, such as 3D printing. The improved medical treatments include greater monitoring and evolution of therapy, culminating in relevant outcomes, more economical medical processes, and the advancement of new prostheses with multiple stimuli-responsive qualities owing to the drive toward intelligent biomaterials. Additionally, developments in smarter biomaterials, combined with reductions and advances in earlier detection, potentially provide greater precision in clinical therapy, fostering the emergence of minimally invasive treatments and reducing the number of deaths due to organ failure or defects.

AI and ML are emerging as transformative tools in biomaterial design, enabling predictive modeling of structure–property–function relationships, high-throughput virtual screening, and multiobjective optimization for tailored performance. Generative algorithms can now propose novel biomaterial architectures with predefined mechanical, chemical, or biological targets, while AI-driven manufacturing optimization accelerates scale-up and reduces costs. When coupled with computational modeling and regulatory data integration, these technologies can significantly shorten development timelines and improve the probability of successful clinical translation. Collectively, the field is converging in developing intelligent adaptive biomaterials capable of addressing complex disease conditions with precision and personalization. Achieving this will require continued innovation in materials science, deeper integration of AI/ML into the design and manufacturing workflow, and robust frameworks to bridge preclinical research with large-scale clinical deployment. As these elements align, the next generation of biomaterials has the potential to redefine medical treatments, improve patient outcomes, and transform global healthcare delivery. This Perspective discusses the design parameters of biomaterials, their clinical applications, and the next-generation biomaterial strategies applied.

## Data Availability

The data supporting the findings of this study are available from the corresponding author upon reasonable request.
